# 初诊慢性髓性白血病患者接受酪氨酸激酶抑制剂治疗现状分析：国内多中心、回顾性真实世界研究

**DOI:** 10.3760/cma.j.cn121090-20231108-00255

**Published:** 2024-03

**Authors:** 小帅 张, 兵城 刘, 新 杜, 龑莉 张, 娜 许, 晓力 刘, 纬明 黎, 海 林, 蓉 梁, 春燕 陈, 健 黄, 云帆 杨, 焕玲 朱, 崚 潘, 晓冬 王, 国辉 李, 卓刚 刘, 延清 张, 振芳 刘, 建达 胡, 春水 刘, 菲 李, 威 杨, 力 孟, 艳秋 韩, 丽娥 林, 震宇 赵, 传清 涂, 彩凤 郑, 炎亮 白, 泽平 周, 苏宁 陈, 惠英 仇, 莉洁 杨, 秀丽 孙, 慧 孙, 励 周, 泽林 刘, 淡瑜 王, 健欣 郭, 丽萍 庞, 庆曙 曾, 晓慧 索, 伟华 张, 媛君 郑, 倩 江

**Affiliations:** 1 北京大学人民医院、北京大学血液病研究所、国家血液系统疾病临床医学研究中心，北京 100044 Peking University People's Hospital, Peking University Institute of Hematology, National Clinical Research Center for Hematologic Disease, Beijing Key Laboratory of Hematopoietic Stem Cell Transplantation, Beijing 100044, China; 2 中国医学科学院血液病医院（中国医学科学院血液学研究所），天津 300020 National Clinical Research Center for Blood Diseases, Institute of Hematology & Blood Diseases Hospital, Chinese Academy of Medical Sciences & Peking Union Medical College, Tianjin 300020, China; 3 深圳市第二人民医院，深圳 518035 The Second People's Hospital of Shenzhen, Shenzhen 518035, China; 4 郑州大学附属肿瘤医院、河南省肿瘤医院、河南省血液病研究所，郑州 450008 Henan Cancer Hospital, The Affiliated Cancer Hospital of Zhengzhou University, Zhengzhou 450008, China; 5 南方医科大学南方医院，广州 510515 Nanfang Hospital, Southern Medical University,Guangzhou 510515, China; 6 华中科技大学同济医学院附属协和医院，武汉 430022 Union Hospital, Tongji Medical College, Huazhong University of Science and Technology, Wuhan 430022, China; 7 吉林大学第一医院，长春 130021 First Hospital of Jilin University, Changchun 130021, China; 8 空军军医大学西京医院，西安 710032 Xijing Hospital, Airforce Military Medical University, Xi'an 710032, China; 9 山东大学齐鲁医院，济南 250012 Qilu Hospital of Shandong University, Jinan 250012, China; 10 浙江大学医学院附属第四医院，杭州 322000 The Fourth Affiliated Hospital of Zhejiang University, Hangzhou 322000, China; 11 四川大学华西医院，成都 610041 Institute of Hematology, West China Hospital, Sichuan University, Chengdu 610041, China; 12 四川省人民医院，成都 610072 Sichuan Academy of Medical Sciences Sichuan Provincial People's Hospital, Chengdu 610072, China; 13 空军军医大学第二附属医院，西安 710038 Xi'an International Medical Center Hospital, Xi'an 710038, China; 14 中国医科大学附属盛京医院，沈阳 110020 Shengjing Hospital of China Medical University, Shenyang 110020, China; 15 哈尔滨医科大学附属第二医院，哈尔滨 150086 The Second Affiliated Hospital of Harbin Medical University, Harbin 150086, China; 16 广西医科大学第一附属医院，南宁 530021 The First Affiliated Hospital of Guangxi Medical University, Nanning 530021, China; 17 福建医科大学附属协和医院，福建省血液病研究所，福建省血液病学重点实验室，福州 350001 Fujian Medical University Union Hospital, Fuzhou 350001, China; 18 南昌大学第一附属医院，南昌大学淋巴肿瘤疾病研究所，南昌 330006 The First Affiliated Hospital of Nanchang University, Nanchang 330006, China; 19 中国医科大学附属盛京医院，沈阳 110020 Shengjing Hospital of China Medical University, Shenyang 110020, China; 20 华中科技大学同济医学院附属同济医院，武汉 430030 Tongji Hospital of Tongji Medical College, Tongji Medical College of Huazhong University of Science and Technology, Wuhan 430030, China; 21 内蒙古医科大学附属医院，呼和浩特 010050 The Affiliated Hospital of Inner Mongolia Medical University, Hohhot 010050, China; 22 海南省人民医院，海口 570311 Hainan General Hospital, Haikou 570311, China; 23 深圳市宝安区人民医院，深圳 518101 Shenzhen Baoan Hospital, Shenzhen University Second Affiliated Hospital, Shenzhen 518101, China; 24 河南省人民医院，郑州 450003 Henan Provincial People's Hospital, Zhengzhou University People's Hospital, Zhengzhou 450003, China; 25 昆明医科大学第二附属医院，昆明 650106 The Second Hospital Affiliated to Kunming Medical University, Kunming 650106, China; 26 苏州大学附属第一医院，江苏省血液研究所，国家血液系统疾病临床医学研究中心，国家卫生健康委员会血栓与止血重点实验室，血液学协同创新中心，苏州 215006 National Clinical Research Center for Hematologic Diseases, Jiangsu Institute of Hematology, the First Affiliated Hospital of Soochow University, Institute of Blood and Marrow Transplantation of Soochow University, Suzhou 215006, China; 27 西安国际医学中心医院，西安 710117 Xi'an International Medical Center Hospital, Xi'an 710117, China; 28 大连医科大学附属第一医院，大连 116011 The First Affiliated Hospital of Dalian Medical University, Dalian 116011, China; 29 郑州大学第一附属医院血液科，郑州 450000 The First Affiliated Hospital of Zhengzhou University, Zhengzhou 450000, China; 30 上海交通大学附属瑞金医院，上海 200025 Shanghai Institute of Hematology, State Key Laboratory of Medical Genomics, National Research Center for Translational Medicine at Shanghai, Ruijin Hospital Affiliated to Shanghai Jiao Tong University School of Medicine, Shanghai 200025, China; 31 华中科技大学协和深圳医院（南山医院），深圳 518000 Huazhong University of Science and Technology Union Shenzhen Hospital, Nanshan Hospital, Shenzhen 518000, China; 32 福建医科大学附属第二医院，泉州 362000 The Second Affiliated Hospital of Fujian Medical University, Quanzhou 362000, China; 33 北京大学深圳医院，深圳 516473 Peking University Shenzhen Hospital, Shenzhen 516473, China; 34 安徽医科大学第一附属医院，合肥 230022 The First Affiliated Hospital of Anhui Medical University, Hefei 230022, China; 35 邯郸市中心医院，邯郸 057150 Handan Central Hospital, Handan 057150, China; 36 山西医科大学第一医院，太原 300012 First Hospital of Shangxi Medical University, Taiyuan 300012, China

**Keywords:** 白血病，髓样，慢性, 酪氨酸激酶抑制剂, 治疗现状, 多中心, 真实世界研究, Leukemia, myeloid, chronic, Tyrosine kinase inhibitor, Treatment status, Multi-centre, Real-world study

## Abstract

**目的:**

回顾性分析国内初诊慢性髓性白血病（CML）患者接受酪氨酸激酶抑制剂（TKI）治疗现状。

**方法:**

回顾性收集来自中国27个省市自治区共77个中心自2006年1月至2022年12月期间确诊、年龄≥18岁、确诊后6个月内接受伊马替尼、尼洛替尼、达沙替尼或氟马替尼作为一线治疗且资料相对完整的CML慢性期（CP）和加速期（AP）病例。分析一线TKI选择、目前用药现状、药物转换及原因，接受TKI治疗反应、结局及其影响因素。

**结果:**

研究最终纳入6 893例接受伊马替尼（4 906例，71.2％）、尼洛替尼（1 157例，16.8％）、达沙替尼（298例，4.3％）或氟马替尼（532例，7.2％）作为一线治疗的成人CML-CP（6 453例，93.6％）和AP（440例，6.4％）患者。所有患者中位随访43（*IQR* 22～75）个月，共1 581例（22.9％）患者由于耐药（1 055例，15.3％）、不耐受（248例，3.6％）、为追求更好疗效（168例，2.4％）、经济或其他原因（110例，1.6％）换药。AP患者换药比例显著高于CP患者（44.1％对21.5％，*P*<0.001），且因耐药而转换治疗的比例也显著更高（75.3％对66.1％，*P*＝0.011）。多因素分析显示，男性、低HGB浓度及ELTS评分中/高危组与CP患者较低的细胞遗传学、分子学反应获得率及较差的结局均相关，高WBC、一线接受第二代TKI治疗与较高的治疗反应获得率相关，初诊时携带Ph^+^附加染色体异常（ACA）与较差的无进展生存（PFS）相关，而Sokal评分中/高危组仅与较低的完全细胞遗传学反应、主要分子学反应获得率和较差的PFS相关；较低的HGB浓度和较大的脾脏与AP患者较低的细胞遗传学和分子学反应获得率相关，一线接受第二代TKI治疗与较高的治疗反应获得率相关，较低的PLT、较高的原始细胞比例和初诊时携带Ph^+^ ACA与较差的无转化生存相关，初诊时携带Ph^+^ ACA与较差的总生存相关。

**结论:**

目前，绝大多数的初诊CP或AP CML患者可长期获益于TKI治疗，获得较好的治疗反应及生存结局。

## Abstract (en)

**Objective:**

To retrospectively analyze the treatment status of tyrosine kinase inhibitors（TKI）in newly diagnosed patients with chronic myeloid leukemia（CML）in China.

**Methods:**

Data of chronic phase（CP）and accelerated phase（AP）CML patients diagnosed from January 2006 to December 2022 from 77 centers, ≥18 years old, and receiving initial imatinib, nilotinib, dasatinib or flumatinib-therapy within 6 months after diagnosis in China with complete data were retrospectively interrogated. The choice of initial TKI, current TKI medications, treatment switch and reasons, treatment responses and outcomes as well as the variables associated with them were analyzed.

**Results:**

6 893 patients in CP（*n*＝6 453, 93.6％）or AP（*n*＝440, 6.4％）receiving initial imatinib（*n*＝4 906, 71.2％）, nilotinib（*n*＝1 157, 16.8％）, dasatinib（*n*＝298, 4.3％）or flumatinib（*n*＝532, 7.2％）-therapy. With the median follow-up of 43（*IQR* 22-75）months, 1 581（22.9％）patients switched TKI due to resistance（*n*＝1 055, 15.3％）, intolerance（*n*＝248, 3.6％）, pursuit of better efficacy（*n*＝168, 2.4％）, economic or other reasons（*n*＝110, 1.6％）. The frequency of switching TKI in AP patients was significantly-higher than that in CP patients（44.1％ *vs* 21.5％, *P*<0.001）, and more AP patients switched TKI due to resistance than CP patients（75.3％ *vs* 66.1％, *P*＝0.011）. Multi-variable analyses showed that male, lower HGB concentration and ELTS intermediate/high-risk cohort were associated with lower cytogenetic and molecular responses rate and poor outcomes in CP patients; higher WBC count and initial the second-generation TKI treatment, the higher response rates; Ph^+^ ACA at diagnosis, poor PFS. However, Sokal intermediate/high-risk cohort was only significantly-associated with lower CCyR and MMR rates and the poor PFS. Lower HGB concentration and larger spleen size were significantly-associated with the lower cytogenetic and molecular response rates in AP patients; initial the second-generation TKI treatment, the higher treatment response rates; lower PLT count, higher blasts and Ph^+^ ACA, poorer TFS; Ph^+^ ACA, poorer OS.

**Conclusion:**

At present, the vast majority of newly-diagnosed CML-CP or AP patients could benefit from TKI treatment in the long term with the good treatment responses and survival outcomes.

2000年后，随着第一代酪氨酸激酶抑制剂（TKI）伊马替尼的问世，慢性髓性白血病（CML）患者的生存期显著延长，生活质量也明显改善[1]–[4]。第二代TKI包括尼洛替尼、达沙替尼和氟马替尼等的应用，使患者获得更快、更深的细胞遗传学和分子学反应，进一步降低疾病进展的风险，改善了患者生存[5]–[7]。目前，国内缺乏大样本量、长期追踪的真实世界研究数据。因此，我们收集了来自中国27个省市自治区共77家中心6 893例初诊CML患者临床资料，回顾性地分析国内CML患者接受TKI治疗现状。

## 病例与方法

一、病例

回顾性收集来自中国27个省市自治区共77个中心自2006年1月至2022年12月期间确诊、年龄≥18岁、确诊后6个月内接受伊马替尼、尼洛替尼、达沙替尼或氟马替尼作为一线治疗且临床资料相对完整的CML慢性期（CP）和加速期（AP）病例资料，包括初诊患者性别、年龄、全血细胞计数、外周血原始细胞及嗜碱性粒细胞比例、脾脏大小（初诊CP患者）、Sokal和ELTS评分、染色体核型、合并症、一线TKI种类和剂量、治疗反应和结局等。

二、诊断分期

患者的诊断和分期参照欧洲白血病网（ELN）推荐的CML诊断和分期标准[8]–[11]。

1. CP：骨髓/外周血原始细胞<15％，且未达AP、急变期（BP）标准。

2. AP：符合下列至少一项指标：①骨髓/外周血原始细胞15％～29％；②原始细胞+早幼细胞≥30％；③外周血嗜碱性粒细胞≥20％；④与治疗无关的持续血小板降低（<100×10^9^/L）；⑤治疗中Ph^+^细胞出现主要途径的附加染色体异常（ACA），包括+8，+Ph［+der（22）t（9;22）（q34;q11）］，i（17）［i（17）（q10）］，+19，ider（22）（q10）t（9;22）（q34;q11）。

3. BP：符合下列至少一项指标：①骨髓/外周血原始细胞≥30％；②髓外原始细胞浸润（除脾脏外）。

三、治疗

纳入研究的患者初始均采用ELN推荐标准剂量[8]–[11]，治疗中根据治疗反应和（或）不良反应等调整TKI剂量或类型。

四、监测

参照ELN推荐进行治疗反应包括血液学、细胞遗传学及分子学监测[8]–[11]。

五、评估指标

1. 治疗反应：根据ELN推荐[8]–[11]，定义完全血液学反应（CHR）、完全细胞遗传学反应（CCyR）、主要分子学反应（MMR）和分子学反应4.5（MR^4.5^）。

2. 结局：无进展生存（PFS）期（初诊CP患者）定义为从开始TKI治疗至首次进展到AP/BP、死亡或末次随访的时间；无转化生存（TFS）期（初诊AP患者）定义为从开始TKI治疗至首次进展至BP或末次随访的时间；总生存（OS）期定义为从开始TKI治疗至死亡或末次随访的时间。所有结局观察均删失至造血干细胞移植。

六、随访

采用门诊或电话联系的方式进行随访，末次随访时间为2023年6月。

七、统计学处理

对于患者基本特征采用描述性统计学分析，计量资料采用*M*（范围）或*M*（*IQR*）表示，计数资料采用例数（％）表示。对连续变量进行两两组间比较时，采用*t*检验或Mann-Whitney *U*非参数检验，对三组及以上的组间比较时采用单因素方差分析法或Kruskal-Wallis非参数检验，对分类变量进行卡方检验或Fisher精确概率法。对于累积治疗反应获得率采用竞争风险模型分析，竞争风险事件为移植或死亡，并应用Fine-Grey检验进行组间比较；对于结局采用Kaplan-Meier生存曲线分析，并应用Log-rank检验进行组间比较。单因素分析*P*<0.2的变量纳入Cox回归模型进行多因素分析。*P*<0.05为差异有统计学意义。采用SPSS 22.0、R 4.0.2及GraphPad Prism 8软件进行统计分析、绘图。

## 结果

一、患者特征

共收集7 735例≥18岁初诊CML-CP和AP病例资料，排除诊断至开始治疗时间>6个月129例、关键临床信息缺失397例、不规律随访或失访262例、非e13a2或e14a2转录本类型54例，最终本研究纳入具有完整人口学信息及临床特征资料的6 893例患者，其中接受伊马替尼（4 906例，71.2％）、尼洛替尼（1 157例，16.8％）、达沙替尼（298例，4.3％）或氟马替尼（532例，7.7％）作为一线治疗的成人CML-CP患者6 453例（93.6％），AP患者440例（6.4％），患者特征见表1。相较于伊马替尼组，接受尼洛替尼或达沙替尼治疗的患者更年轻（*P*<0.001），初诊时WBC、外周血原始细胞和嗜碱性粒细胞比例更高（*P*值均<0.001），而HGB浓度更低（*P*＝0.047），且有更高比例的Sokal或ELTS评分中高危和AP患者（*P*<0.001）。接受氟马替尼治疗的患者相较于伊马替尼组更年长（*P*＝0.002），初诊时有高比例的患者伴有合并症（*P*＝0.009）。接受达沙替尼治疗的患者相较于尼洛替尼组有更高比例的Sokal或ELTS评分中高危和AP（*P*<0.001）。

**表1:** 6 893例初诊接受酪氨酸激酶抑制剂的慢性髓性白血病患者基线特征

变量	患者总体（6 893例）	伊马替尼组（4 906例）	尼洛替尼组（1 157例）	达沙替尼组（298例）	氟马替尼组（532例）	统计量	*P*值
男性[例（%）]	4 186（60.7）	2 957（60.3）	721（62.3）	180（60.4）	328（61.7）	1.854	0.603
年龄[岁，*M*（范围）]	42（18~89）	42（18~87）	38（18~77）	40（18~89）	45（18~80）	28.499	< 0.001
分期与危险度分层[例（%）]							
慢性期	6 453（93.6）	4 640（94.6）	1 053（91.0）	255（85.6）	505（94.9）	54.540	< 0.001
Sokal危险度^a^						23.225	0.001
低危	2 643（41.0）	1 913（41.2）	451（42.8）	82（32.2）	197（39.0）		
中危	1 904 29.5）	1 362（29.4）	316（30.0）	71（27.8）	155（30.7）		
高危	1 027（15.9）	694（15.0）	169（16.0）	59（23.1）	105（20.8）		
未知	879（13.6）	671（14.4）	117（11.2）	43（16.9）	48（9.5）		
ELTS危险度^a^						29.669	< 0.001
低危	3 788（58.7）	2 738（59.0）	634（60.2）	124（48.6）	292（57.8）		
中危	1 373（21.3）	964（20.8）	232（22.0）	55（21.6）	122（24.2）		
高危	413（6.4）	267（5.8）	70（6.6）	33（12.9）	43（8.5）		
未知	879（13.6）	671（14.4）	117（11.2）	43（16.9）	48（9.5）		
加速期	440（6.4）	266（5.4）	104（9.0）	43（14.4）	27（5.1）	54.540	< 0.001
WBC[×10^9^/L，*M*（范围）]	126.8（1.5~874.4）	120.0（1.7~874.4）	150.0（1.5~754.7）	158.3（2.1~770.0）	130.8（3.8~791.7）	8.286	< 0.001
HGB[g/L，*M*（范围）]	112（18~1 151）	113（18~340）	109（43~1 151）	107（33~345）	112（45~186）	2.646	0.047
PLT[×10^9^/L，*M*（范围）]	424（5~6 111）	413（6~6 111）	400（16~5 283）	427（5~2 813）	447（30~2 500）	1.981	0.114
外周血原始细胞[%，*M*（范围）]	1.0（0~29.0）	1.0（0~29.0）	1.0（0~27.0）	1.0（0~27.0）	1.0（0~20.0）	11.072	< 0.001
外周血嗜碱性粒细胞[%，*M*（范围）]	4.6（0~86.1）	4.1（0~77.0）	5.0（0~63.0）	5.0（0~86.1）	4.0（0~52.0）	16.236	< 0.001
初诊脾脏肋缘下厘米数[cm，*M*（范围）]	3.0（0~32.0）	3.0（0~32.0）	3.0（0~25.0）	3.5（0~25.0）	3.0（0~23.0）	3.215	0.022
Ph^+^ ACA[例（%）]	228（3.3）	151（3.1）	47（4.1）	6（2.0）	24（4.5）	6.190	0.103
高危ACA[例（%）]	90（1.3）	58（1.2）	24（2.1）	2（0.6）	6（1.1）	6.043	0.104
合并症[例（%）]	1 630（23.6）	1 152（23.5）	248（21.4）	78（26.2）	152（28.6）	11.410	0.010
随访时间[月，*M*（IQR）]	43（22~75）	50（29~84）	32（16~60）	29（15~52）	19（11~24）	344.177	< 0.001

注 ^a^仅在慢性期患者中计算Sokal和ELTS评分并分层，比例为占慢性期患者的百分比；ACA：附加染色体异常

所有患者中位随访43（*IQR* 22～75）个月，共1 581例（22.9％）患者由于耐药（1 055例，15.3％）、不耐受（248例，3.6％）、为追求更好疗效（168例，2.4％）、经济或其他原因（110例，1.6％）换药。截至末次随访，3 641例（52.8％）患者服用伊马替尼，1 321例（19.2％）服用尼洛替尼，735例（10.7％）服用达沙替尼，763例（11.1％）服用氟马替尼，108例（1.6％）参与临床试验，31例（0.4％）接受干扰素±羟基脲治疗，106例（1.5％）接受化疗，91例（1.3％）患者因怀孕（8例，0.1％）或追求无治疗缓解（83例，1.2％）而停药，97例（1.4％）因疾病进展（94例，1.3％）或主动选择（3例，0.1％）移植（图1A）。

CP和AP患者中位随访时间分别为46（*IQR* 25～78）个月和42（*IQR* 19～72）个月（*P*＝0.174），患者治疗过程中的换药情况及原因见图1B。AP患者换药比例显著高于CP患者（44.1％对21.5％，*P*<0.001），且因耐药而转换治疗的比例也显著更高（75.3％对66.1％，*P*＝0.011）。

伊马替尼、尼洛替尼、达沙替尼和氟马替尼组患者中位随访时间分别为50（*IQR* 29～84）、32（*IQR* 16～60）、29（*IQR* 15～52）和19（*IQR* 11～24）个月（*P*<0.001），伊马替尼组最长，氟马替尼组最短，而尼洛替尼和达沙替尼组差异无统计学意义（*P*＝0.243），各组患者换药情况及原因见图1C。

**图1:**
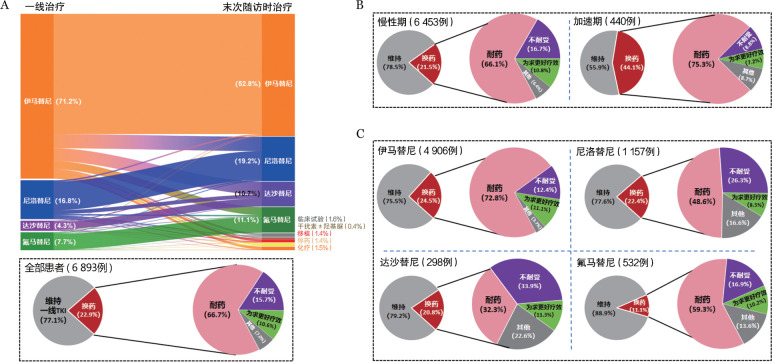
慢性髓性白血病患者酪氨酸激酶抑制剂治疗转换现状及转换原因 A 所有患者；B 慢性期与加速期患者；C 伊马替尼、尼洛替尼、达沙替尼和氟马替尼治疗组

二、CP患者治疗反应、结局及其影响因素

6 453例CP患者中位随访46（*IQR* 25～78）个月，6 350例（98.4％）在治疗3个月内获得CHR，5 889例（91.3％）获得CCyR，4 879例（75.6％）、2 919例（45.2％）分别获得MMR和MR^4.5^。1 477例（22.9％）在治疗过程中至少经历1次治疗失败，其中，305例（4.7％）疾病进展至AP（191例，3.0％）或BP（114例，1.8％），114例（1.8％）患者死于疾病进展（91例，1.4％）或其他原因（23例，0.4％）。7年CCyR、MMR和MR^4.5^累积获得率分别为96.4％（95％*CI* 94.1％～98.5％）、86.2％（95％*CI* 83.0％～89.7％）和64.8％（95％*CI* 61.3％～67.2％）。7年PFS率和OS率分别为93.1％（95％*CI* 87.5％～99.4％）和97.9％（95％*CI* 94.3％～100％）。CP患者接受伊马替尼或第二代TKI的治疗反应及结局见表2、图2A。

**表2:** 初诊慢性髓性白血病慢性期和加速期患者接受伊马替尼或第二代TKI的治疗反应及结局

组别	例数	随访时间[月，*M*（*IQR*）]	治疗反应［％（95％ *CI*）］				生存结局［％（95％ *CI*）］		
			时间（年）	CCyR	MMR	MR^4.5^	时间（年）	PFS	OS
慢性期	4 640								
伊马替尼	1 813	46（25~78）	7	94.1（92.5~96.7）	83.4（79.0~87.3）	60.2（56.1~64.8）	7	90.8（86.4~94.5）	92.1（88.3~96.8）
第二代TKI		30（16~59）	5	97.3（95.0~99.6）	88.7（84.0~92.2）	65.7（61.2~69.4）	5	95.2（93.1~97.6）	96.5（94.0~98.2）
加速期									
伊马替尼	266	52（27~85）	7	85.5（83.7~87.2）	80.1（77.6~83.3）	58.2（51.3~65.9）	7	89.5（85.2~93.6）	88.8（82.5~94.0）
第二代TKI	174	37（18~49）	4	93.2（90.9~96.7）	85.6（81.7~89.4）	62.3（52.5~71.8）	4	88.8（82.5~94.0）	89.8（81.4~97.2）

注 TKI：酪氨酸激酶抑制剂；CCyR：完全细胞遗传学反应；MMR：主要分子学反应；MR^4.5^：分子学反应4.5；PFS：无进展生存；OS：总生存

**图2:**
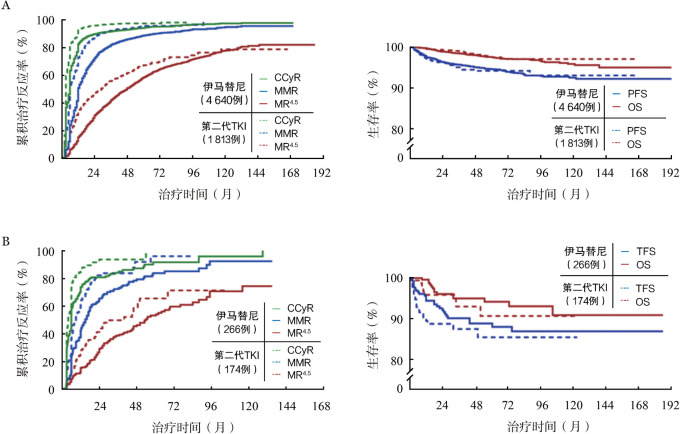
初诊慢性髓性白血病慢性期和加速期患者治疗反应及结局 A 慢性期；B 加速期 注 CCyR：完全细胞遗传学反应；MMR：主要分子学反应；MR^4.5^：分子学反应4.5；PFS：无进展生存；OS：总生存；TFS：无转化生存

为探索初诊CP患者治疗反应和结局的影响因素，将患者性别、初诊时WBC、HGB浓度、外周血嗜碱性粒细胞比例、Sokal和ELTS危险度分组、Ph^+^ACA、合并症及一线TKI进行单因素分析，多因素分析发现，男性、低HGB浓度及ELTS评分中/高危组与CP患者较低的细胞遗传学、分子学反应获得率及较差的结局均相关，高WBC及一线接受尼洛替尼、达沙替尼或氟马替尼治疗与较高的治疗反应获得率相关，初诊时携带Ph^+^ ACA与较差的PFS相关，而Sokal评分中/高危组仅与较低的CCyR、MMR获得率和较差的PFS相关，与MR^4.5^、PFS和OS无显著相关性（表3）。ELTS评分危险度与CP患者获得TKI治疗反应和结局的相关性见图3。

**表3:** 初诊慢性髓性白血病慢性期患者酪氨酸激酶抑制剂（TKI）治疗反应及结局的多因素分析

变量	CCyR		MMR		MR^4.5^		PFS		OS	
	*HR*（95%*CI*）	*P*值	*HR*（95%*CI*）	*P*值	*HR*（95%*CI*）	*P*值	*HR*（95%*CI*）	*P*值	*HR*（95%*CI*）	*P*值
男性	0.9（0.8 ~1.0）	<0.001	0.8（0.8 ~0.9）	<0.001	0.8（0.7 ~0.9）	<0.001	1.4（1.0 ~1.7）	0.021	1.3（1.0 ~1.7）	0.036
WBC ^a^	0.9（0.9 ~0.9）	<0.001	0.8（0.8 ~0.9）	<0.001	0.8（0.7 ~0.8）	<0.001				
HGB ^b^	1.0（1.0 ~1.0）	<0.001	1.0（1.0 ~1.0）	<0.001	1.0（1.0 ~1.0）	0.008	0.9（0.8 ~0.9）	<0.001	0.9（0.8 ~0.9）	<0.001
Sokal危险度		<0.001		0.016				0.046		
低危（参考）										
中危	0.8（0.7 ~0.9）	0.006	0.8（0.7 ~0.9）	0.010			1.4（1.2 ~1.7）	0.014		
高危	0.7（0.6 ~0.8）	<0.001	0.8（0.7 ~0.9）	0.009			2.0（0.9 ~2.6）	0.125		
未知	0.9（0.5 ~1.1）	0.472	0.9（0.7 ~1.4）	0.325			0.8（0.4 ~1.3）	0.303		
ELTS危险度		<0.001		<0.001		<0.001		<0.001		<0.001
低危（参考）										
中危	0.8（0.8 ~0.9）	<0.001	0.8（0.8 ~0.9）	<0.001	0.8（0.7 ~0.9）	<0.001	1.9（1.5 ~2.6）	<0.001	2.0（1.5 ~2.6）	<0.001
高危	0.7（0.6 ~0.8）	<0.001	0.7（0.6 ~0.8）	<0.001	0.7（0.6 ~0.9）	<0.001	3.6（2.6 ~5.1）	<0.001	3.9（2.8 ~5.5）	<0.001
未知	0.9（0.8 ~1.0）	0.285	0.9（0.8 ~1.0）	0.016	0.7（0.6 ~0.8）	<0.001	0.6（0.3 ~1.1）	0.120	0.6（0.3 ~1.1）	0.103
Ph^+^ ACA							1.8（1.1 ~3.0）	0.016		
合并症							1.3（1.0 ~1.7）	0.100		
一线第二代TKI治疗（伊马替尼为参考）	1.6（1.5 ~1.7）	<0.001	1.9（1.7 ~2.0）	<0.001	1.6（1.5 ~1.8）	<0.001	0.7（0.4 ~1.1）	0.180		

注 ^a^ WBC每增加100×10^9^/L的风险比；^b^ HGB浓度每增加10 g/L的风险比；CCyR：完全细胞遗传学反应；ELTS：CML欧洲长期生存评分；MMR：主要分子学反应；MR^4.5^：分子学反应4.5；PFS：无进展生存；OS：总生存；Ph^+^ ACA：Ph染色体阳性附加染色体异常

**图3:**
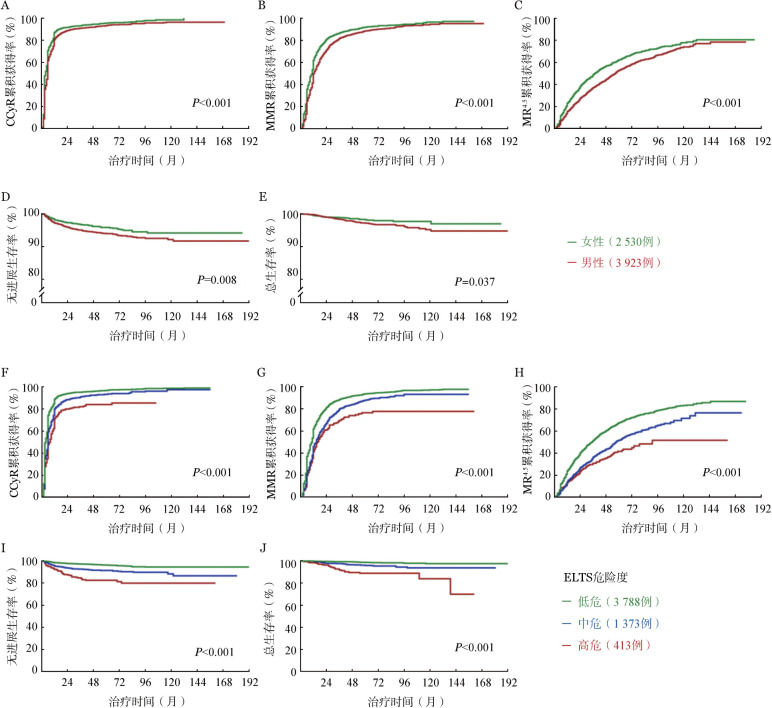
性别（A～E）和ELTS危险度分层（F～J）对初诊慢性髓性白血病慢性期患者治疗反应及结局的影响 注 CCyR：完全细胞遗传学反应；MMR：主要分子学反应；MR^4.5^：分子学反应4.5

三、AP患者治疗反应、结局及其影响因素

440例初诊AP患者中位随访时间为42（*IQR* 19～72）个月，409例（92.9％）在治疗3个月内获得CHR，368例（83.6％）获得CCyR，301例（68.4％）、198例（45.0％）分别获得MMR和MR^4.5^。148例（33.6％）在治疗过程中至少经历1次治疗失败，其中，43例（9.8％）疾病进展至BP，42例（9.5％）患者死于疾病进展（37例，8.4％）或其他原因（7例，1.1％）。6年CCyR、MMR和MR^4.5^累积获得率分别为89.8％（95％*CI* 87.2％～91.5％）、80.2％（95％*CI* 77.6％～83.4％）和59.1％（95％*CI* 54.4％～64.9％）。6年TFS率和OS率分别为87.0％（95％*CI* 83.6％～91.4％）和92.6％（95％*CI* 89.1％～95.9％）。AP患者接受伊马替尼或第二代TKI的治疗反应及结局见表2、图2B。

为探索初诊AP患者治疗反应和结局的影响因素，将患者性别、初诊时年龄、WBC、HGB浓度、PLT、原始细胞和外周嗜碱性粒细胞比例、肋缘下脾脏大小、Ph^+^ ACA、合并症及一线TKI进行单因素分析，多因素分析发现，初诊时较低的HGB浓度和较大的脾脏与较低的细胞遗传学和分子学反应获得率相关。此外，相较于伊马替尼，一线接受尼洛替尼、达沙替尼或氟马替尼治疗与较高的治疗反应获得率相关（表3）。较低的PLT、较高的原始细胞比例和初诊时携带Ph^+^ ACA与较差的TFS相关，初诊时携带Ph^+^ ACA与较差的OS相关（表4）。

**表4:** 初诊慢性髓性白血病加速期患者酪氨酸激酶抑制剂（TKI）治疗反应及结局的多因素分析

变量	CCyR		MMR		MR^4.5^		TFS		OS	
	*HR*（95%*CI*）	*P*值	*HR*（95%*CI*）	*P*值	*HR*（95%*CI*）	*P*值	*HR*（95%*CI*）	*P*值	*HR*（95%*CI*）	*P*值
男性			0.8（0.6~1.0）	0.091					2.5（1.0~6.6）	0.056
年龄 ^a^			0.9（0.8~1.0）	0.057					1.3（1.0~1.8）	0.061
HGB ^b^	1.1（1.0~1.2）	0.001	1.1（1.0~1.2）	0.001	1.1（1.0~1.2）	0.013				
PLT ^c^							0.9（0.8~1.0）	0.013		
原始细胞比例 ^d^							1.0（1.0~1.1）	0.010		
脾脏大小	0.9（0.9~0.9）	0.021	0.9（0.9~0.9）	0.002	0.9（0.9~0.9）	0.008				
Ph^+^ ACA							3.6（1.6~8.5）	0.003	3.6（1.2~10.8）	0.022
合并症							1.9（1.0~3.7）	0.058	2.2（0.9~5.2）	0.076
一线第二代TKI治疗（伊马替尼为参考）	1.6（1.2~2.1）	0.001	2.0（1.5~2.7）	<0.001	1.7（1.1~2.5）	0.009				

注 ^a^ 年龄每增加10岁的风险比；^b^ HGB浓度每增加10 g/L的风险比；^c^ PLT每增加100×10^9^/L的风险比；^d^ 以初诊时骨髓或外周最高原始细胞比例记录；CCyR：完全细胞遗传学反应；MMR：主要分子学反应；MR^4.5^：分子学反应4.5；TFS：无转化生存；OS：总生存；ELTS：CML欧洲长期生存评分；Ph^+^ ACA：Ph染色体阳性附加染色体异常

## 讨论

本研究是一项国内多中心回顾性研究，是迄今为止最大样本量的长期追踪的CML数据。我们发现，中国CML患者的一线TKI选择仍以伊马替尼为主，TKI中位治疗近4年后，仍有半数患者服用伊马替尼。22.9％的患者在治疗过程中至少经历1次治疗转换，半数以上是因为耐药而转换治疗。在TKI时代，大部分初诊CP和AP患者都能获得较好的治疗反应，约60％的患者获得深层分子学反应，超过90％的患者长期生存。

近十年来，随着第二代TKI的问世、仿制药上市、医保政策改善以及人们生活水平提高，TKI药物的可及性普遍提高，选择第二代TKI作为一线治疗的患者比例增加[3]–[4],[12]–[14]，在我们的研究中，28.8％的患者接受一线第二代TKI包括尼洛替尼、达沙替尼和氟马替尼治疗，相较于伊马替尼，接受第二代TKI治疗可获得更快、更深层细胞遗传学和分子学反应，尽管在生存结局上相当。在各种TKI均可及的情况下，医师可以根据CML患者的疾病特征、合并症、治疗目标和意愿等，制定更加个体化的治疗方案，使患者获益。

本研究结果显示，22.9％的患者在治疗过程中至少经历1次治疗转换，换药患者中66.7％因为耐药、15.7％因为不耐受而转换治疗，初诊AP患者中因耐药而转换治疗的比例显著高于CP患者。尽管目前认为初诊AP患者总体预后较好，但有研究报道其早期分子学反应及无失败生存率仍差于CP患者[15]–[17]，与本研究结果相似。

本研究结果还显示，不同TKI治疗组之间的换药比例有差异：氟马替尼组换药比例相较于伊马替尼、尼洛替尼和达沙替尼组更低，其主要原因可能是当前氟马替尼治疗组随访时间较短。在相似的随访时间下，尽管尼洛替尼和达沙替尼组患者的总体治疗转换率相当（22.4％对20.8％），但达沙替尼组患者因为不耐受而转换治疗的患者比例略高于尼洛替尼组（33.9％对26.3％），这一结论仍需在前瞻性、随机对照试验中进一步验证。

在绝大部分CML患者得益于TKI治疗获得长期生存背景下，识别其中的高危人群尤为重要。如今，Sokal和ELTS评分是临床上对初诊CP患者最常用的危险度分层工具。相较于Sokal评分，ELTS评分对治疗反应和结局的预测效能更佳，尤其是在第二代TKI治疗人群中[3],[18]。本研究在国内多中心大样本量人群中进一步证实，ELTS评分的预测效能显著优于Sokal评分。此外，与既往研究结果一致，本研究也证实男性及初诊时低HGB浓度与较差的治疗反应和结局均显著相关[3]–[4],[19]–[20]。

本研究是一项真实世界的回顾性临床研究，存在以下局限性：①由于尼洛替尼、达沙替尼和氟马替尼上市时间相对较晚，纳入研究的患者数量有限，且相较于伊马替尼治疗组随访时间也显著更短，未来需要更大样本量、更长随访时间的研究，予以进一步探索和验证；②因数据来自多家中心，不同实验室采用的检测结果可能存在差异，导致对TKI治疗反应的评估难以精确到MR^4^及以上的深层分子学反应；③由于本研究中部分来自其他中心的患者监测、随访不规范，原始数据保留情况较差，导致部分患者无法准确得知其是否在服药期间发生过血液学或非血液不良反应及其严重程度，因此，本文并未呈现TKI相关不良反应数据。

总之，我们通过分析来自国内多中心的数千例初诊CML患者数据，呈现了中国当前CML的用药现状及总体治疗结果，显示绝大多数的初诊CP或AP CML患者可以长期获益于TKI治疗。
